# Comparative Evaluation of Forced Swim Test and Tail Suspension Test as Models of Negative Symptom of Schizophrenia in Rodents

**DOI:** 10.5402/2012/595141

**Published:** 2012-01-12

**Authors:** Manavi Chatterjee, Manoj Jaiswal, Gautam Palit

**Affiliations:** Neuropharmacology Unit, Division of Pharmacology, Central Drug Research Institute, CSIR, Uttar Pradesh, Lucknow 226001, India

## Abstract

Previous studies have shown that the administration of NMDA antagonist can induce negative symptoms of schizophrenia which can be tested through the enhanced immobility observed in the forced swim test (FST). In the present study, we have compared the effects of acute as well as chronic administration of a noncompetitive NMDA receptor antagonist, ketamine on FST, and another behaviour despair model, tail suspension test (TST). Our observations suggest that chronic ketamine administration induced a state of enhanced immobility in FST, but such findings were not replicated in the TST model. Further, in FST, treatment with clozapine reverses the ketamine-induced immobility in mice, whereas it enhances the immobility duration in the TST model. However, haloperidol showed no protective effects in both models. The data suggests that although both of these tests show common behavioural measure of feeling despair, however, the underlying pathophysiology seems to be different. Hence, forced swim test but not tail suspension test can be used as a model of negative symptom of psychosis in mice.

## 1. Introduction

Schizophrenia is perhaps the most devastating and enduring psychotic disorder affecting as many as 1% of the population worldwide, with a similar prevalence between the sexes and throughout diverse cultures and geographic areas [1].

Patients may present with positive symptoms (such as conceptual disorganization, delusions, hallucinations, hyperactivity, or stereotypy behaviour) or negative symptoms (social withdrawal, loss of function, anhedonia, decreased emotional expression, impaired concentration, and diminished social engagement). The latter predominate in one-third of the schizophrenic population and are associated with a poor long-term outcome and a poor response to drug treatment [2].

 The treatment of schizophrenic patients with a typical antipsychotic, haloperidol, has been successful against positive symptoms but has a poor effect on primary negative symptoms [3], and clozapine, an atypical antipsychotic, apparently shows a partial effect on negative symptoms [4, 5]. Very little progress has been made in developing new antipsychotics with a combined effect on positive and primary negative symptoms, and part of the reason for this failure is the limited number of appropriate animal models that can mimic these symptoms and can be used for drug screening [6, 7]. Consequently, it is of great importance to determine whether and how antipsychotics improve negative symptoms in patients with schizophrenia, and also the effect of the antipsychotics can be predicted in animal models. Thus, in our present study, we have compared the effects of NMDA receptor antagonist, ketamine in tail suspension test and forced swim test model, and validated it by using classical antipsychotics, in an attempt to develop a model for negative symptoms of psychosis using ketamine.

## 2. Materials and Methods

### 2.1. Drugs

Ketamine injections were purchased from Ranbaxy, India. Clozapine and haloperidol were procured from Sigma-Aldrich, UK.

### 2.2. Animals

Experimental protocols were approved by our Institutional Ethical Committee following the guidelines of Committee for the Purpose of Control and Supervision of Experiments on Animals (CPCSEA) which complies with international norms of Indian National Science Academy (INSA). Male Swiss mice weighing 20–25 g were employed in the study. All animals were housed in polyacrylic cage in environmentally controlled rooms (temperature 24–27°C and humidity 60–65% with 12 : 12 light : dark cycle). Food was provided in the form of dry pellets, and water was given *ad libitum*.

### 2.3. Treatment Schedule

Ketamine injections were diluted in saline. Clozapine and haloperidol were dissolved in minimal acetic acid and titrated to pH 6.0 using NaOH.

Mice were divided into different groups, and each group consisted of 8 mice. The five treatment groups were vehicle + saline (VS), vehicle + ketamine (VK), haloperidol + ketamine (HK), and clozapine + ketamine (CK). The experiments were carried out in three phases.

**Phase 1 : (acute study)**

The mice were pretreated with either vehicle or antipsychotic drug 30 min prior to the administration of ketamine (100 mg/kg, i.p.).

**Phase 2 : (chronic study)**

The mice were pretreated with either vehicle or antipsychotic drug 30 min prior to the administration of ketamine (100 mg/kg/day, i.p.) for 10 days. In the appropriate test sessions, the minimal acetic acid solution was used as control vehicle solution.

**Phase 3 : (withdrawal study)**

The antipsychotics and ketamine were withdrawn after 10 days of repeated treatment, and the animals were subjected to the withdrawal study, where no treatments were given.

### 2.4. Behavioural Studies

#### 2.4.1. Tail Suspension Test

The tail suspension test (TST) was performed according to the method described previously [8]. The mice were individually suspended in the hook of the tail suspension test box, 60 cm above the surface of table with an adhesive tape placed 1 cm away from the tip of the tail. After 1 min acclimatization, immobility duration was recorded for 5 minutes from side view using small fire-wire cameras and the ANY-maze software (Stoelting Co., Wood Dale, IL). Mice were considered immobile only when they hung passively and were completely motionless.

#### 2.4.2. Forced Swimming Test

Forced swimming test in mice, established previously [9], is a behavioural despair test. The mice were placed individually in glass cylinders (20 cm height, 10 cm diameter) containing 10 cm depth of water at 25°C. After 5 minutes, the animals were removed from water, dried, and returned back to their home cages. They were again placed in the cylinder 24 hr later, and after the initial 1 min acclimatization period, the total duration of immobility was measured for 5 mins. Mice were considered to be immobile when they were floating motionless. The duration of swimming was recorded for 5 min from side-view using small fire-wire cameras and the ANY-maze software (Stoelting Co., Wood Dale, IL).

## 3. Statistical Analysis

Data were expressed as mean + SEM. Analysis was performed with Prism version 3.0 software using one-way analysis of variance (ANOVA) followed by Neumann Keul's multiple comparison test. *P* < 0.05 was considered to be statistically significant.

## 4. Results

### 4.1. Acute Studies

**Figure 1:**
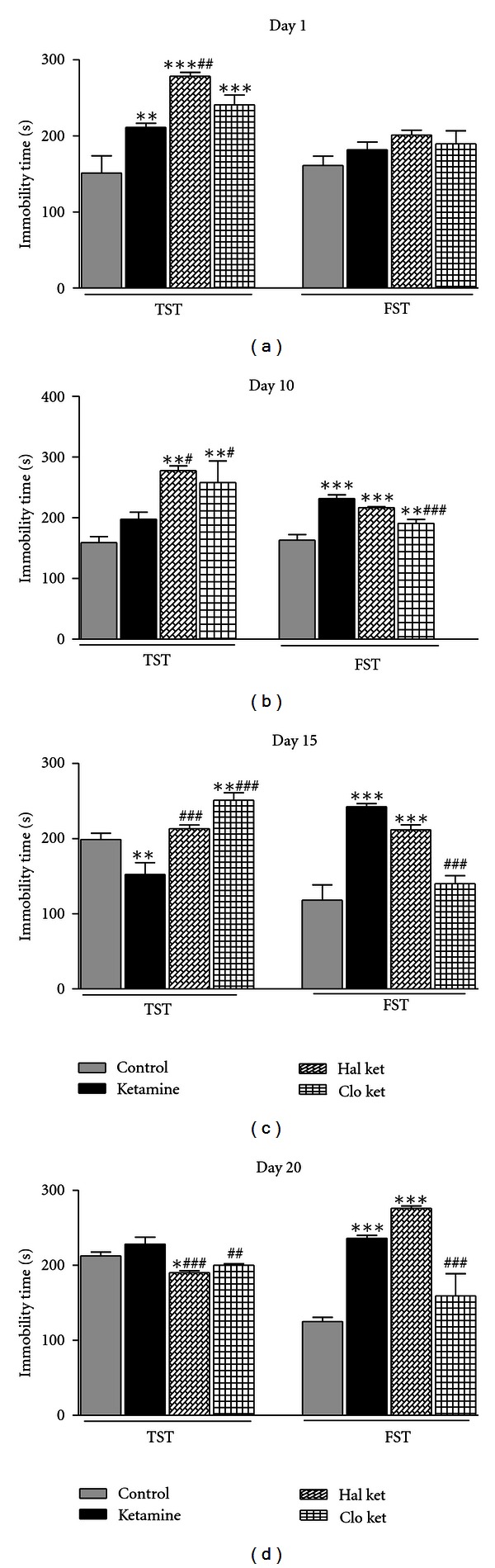
Day-dependent effect of ketamine administration on enhanced immobility in the tail suspension test and forced swim test in mice. Bar diagram representing the day-dependent study on the effects of chronic treatment of ketamine and antipsychotic drugs, and their withdrawal, on the immobility duration (in secs) of mice in tail suspension test and forced swim test. Results are represented as mean ± SEM with *n* = 8 in each group. **P < 0.01, ***P < 0.001 when compared with control group; ^##^ *P* < 0.01, ^###^ *P* < 0.001 when compared with the ketamine-induced group (ket).

To assess whether acute ketamine can induce immobility in mice in the tail suspension and swim despair paradigm or not, we subjected the animals to acute ketamine treatment with single dose of 100 mg/kg, i.p., 30 mins prior to the trials. We have observed that acute treatment with ketamine significantly enhanced the immobility time by 39.7%, in the tail suspension test as compared to vehicle-treated controls. This effect of enhanced immobility was not observed in the forced swim test. To further validate the model, we administered both haloperidol and clozapine, 30 mins prior to ketamine administration, and observed their effects in immobility in both experiments. Our observations revealed that both the antipsychotics enhanced the immobility duration significantly with respect to control by 84.1% (*P* < 0.001) and 58.94% (*P* < 0.001), whereas it increased the immobility duration by 31.75% (*P* < 0.01) and 14.09% with respect to ketamine-treated groups in the tail suspension model. However, no such changes were observed in the forced swim test (Figure 1(a)). These findings suggest that the acute ketamine-induced immobility in TST does not show predictive validity to study the negative symptoms of schizophrenia.

### 4.2. Chronic Studies

Since, our acute ketamine regimen did not sufficiently induce immobility in the forced swim test, we shifted to a chronic ketamine treatment regime and studied the effect on immobility duration in both tail suspension and forced swim models. Our observations indicate that chronic ketamine treatment for 10 days did not cause any significant enhancement of immobility duration, in the TST model. Moreover, both antipsychotics induced significant enhancement in the immobility duration by 74.71% (*P* < 0.01) and 62.26% (*P* < 0.01) with respect to control (Figure 1(b)).

However, in the forced swim test, we have found that repeated ketamine treatment for 10 days significantly enhanced the immobility duration by 41.9%. Also pretreatment with the typical antipsychotic haloperidol could not attenuate the ketamine-induced immobility time, whereas clozapine-pretreated mice showed sufficient protection against ketamine-induced immobility by 17.71% (*P* < 0.001). The above findings revealed that amongst both TST and FST, the latter showed predictive validity for studying the negative symptoms of schizophrenia (Figure 1(b)).

### 4.3. Withdrawal Studies

We further evaluated the persistence of effects of ketamine in TST and FST after the withdrawal of drugs on both the 15th day (5 days after withdrawal) and the 20th day (10 days after withdrawal).

#### 4.3.1. 15th Day

In tail suspension test, the withdrawal of ketamine after 5 days caused reduction in the immobility duration by 23.23%. The group treated with haloperidol was not significantly different from the control group but showed a significant enhancement in the immobility time when compared with ketamine-treated groups. Clozapine significantly enhanced the immobility time by 26.28% (*P* < 0.01) and 64.69% (*P* < 0.001) as compared to control- and ketamine-treated groups, respectively (Figure 1(c)).

However, in the forced swim test, the immobility induced by ketamine persisted even after 5 days of drug withdrawal. The immobility time was significantly different from control and showed an increase of approximately 105.3% (*P* < 0.001). Further, the group treated with haloperidol did not normalize the ketamine-induced effects even after withdrawal. However, the clozapine could significantly reduce the ketamine-induced immobility duration by 42.20% (*P* < 0.001) (Figure 1(c)).

#### 4.3.2. 20th Day

In an attempt to check the duration of persistence of ketamine-induced effects, we carried out our experiments till the 20th day (i.e., 10 days after withdrawal). As found earlier, in tail suspension test, no significant change was observed in the immobility duration, after 10 days of withdrawal of ketamine, whereas the haloperidol and the clozapine groups showed decrease in immobility duration by 10.58% (*P* < 0.05) and 5.88% with respect to control (Figure 1(d)).

In the forced swim test, ketamine showed persistent immobility even after 10 days of withdrawal. The enhancement of immobility by 88.8% was significant with respect to control. This effect was not normalized in haloperidol groups but was significantly reduced by 32.5% (*P* < 0.001) in clozapine groups (Figure 1(d)).

## 5. Discussion

Typically, the negative symptoms of schizophrenia include social and emotional withdrawal, avolition and anhedonia. Given the close similarity between the negative symptoms of schizophrenia and the core symptoms of depression, a first approach to modelling negative symptoms in schizophrenia should be to use classical animal models for depression, such as the tail suspension test (TST) or the forced swim test (FST). Also, since both of these models are behavioural despair tests, they were also assumed to represent lack of motivational behaviour frequently observed in schizophrenic patients. Therefore, the effect of ketamine treatment was observed in TST and FST.

In our study, we aimed to compare the acute, chronic, and withdrawal effects of ketamine and antipsychotic drugs on tail suspension test and forced swim test to evaluate their abilities as the model for studying the negative symptoms of schizophrenia.

Our observations showed that single-dose injection of ketamine enhanced the immobility duration in mice in the TST but not FST. This may imply that ketamine induces a certain neurochemical abnormality which causes the mice to remain immobile in the TST and enter in a state of despair. This effect was further aggravated with antipsychotic treatments in the TST and not FST, which may also indicate that TST is more sensitive to acute neurochemical changes than FST.

Chronic treatment with ketamine for 10 days showed no significant effects in the TST, while pretreatment with antipsychotics further enhanced the immobility duration, instead of attenuating it, while, in the case of FST, chronic ketamine treatment enhanced immobility duration which persisted even after drug withdrawal for up to 10 days (i.e., 20th day of the experiment). The immobility enhancement was found to be attenuated by clozapine, whereas haloperidol failed to improve the symptoms, which in turn verifies that typical antipsychotic drugs are ineffective in improving the negative symptoms of psychosis. Our observations demonstrating exacerbation of schizophrenic symptomatology are consistent with previous observations of PCP's effects in schizophrenia [10, 11], this behavioural change being regarded as avolition which is one of the negative symptoms of schizophrenia [10].

An increasing number of investigations have centred on the role of monoamines in the pathogenesis of negative symptoms. There is evidence that serotonergic (5-HTergic) dysfunction is associated with negative symptoms in schizophrenia [12–14]. It is well established that atypical antipsychotics, such as clozapine and risperidone, which are potent serotonin (5-HT) receptor antagonists, improve negative symptoms in schizophrenia [15–17]. Thus, it appears that 5-HTergic functions are overactive in negative symptoms of schizophrenia. However, the mechanisms of negative symptom-like behavioural changes induced by NMDA receptor antagonists have yet to be elucidated. It has been suggested that a disturbance in the balance of 5-HT and dopamine-mediated neurotransmission underlies schizophrenia [13]; an increased 5-HTergic activity relative to dopaminergic activity might lead to negative symptoms. This hypothesis is based on the evidence that 5-HT-dopamine antagonists such as risperidone and clozapine improve negative symptoms. Commonly used antipsychotics that potently block dopamine receptors such as haloperidol and chlorpromazine are more effective in treating positive symptoms manifested in chronic schizophrenic patients [18], whereas negative symptoms such as the flattening of affect and avolition are less responsive. Thus, the overactivity of presynaptic 5-HTergic systems in the prefrontal cortex may be responsible for the expression of the enhancement of immobility in mice pretreated with NMDA antagonists repeatedly.

In our study, induction of immobility by repeated ketamine treatment in the FST but not in the TST implies that both of these models might follow different pathophysiological mechanisms. Excessive release of serotonin leads to overactivity of the 5HT2A system, which leads to enhanced immobility in the FST model but induces hyperactivity in the TST model. Further, blockade of DA receptors by haloperidol aggravates the symptoms in both FST and TST. However, clozapine pretreatment attenuates ketamine-induced immobility in the FST model but shows conflicting results in the TST system. This pharmacological evaluation indicates that dopamine functioning is a necessity for performance of mice in the FST, whereas both serotonergic and dopaminergic systems are involved in TST model. 

Therefore, our studies show that though both of these models are similar in face behavioural symptoms, that is, symptoms of despair, they have different underlying mechanisms of inducing immobility. In addition, our findings also indicate that only forced swim test model but not tail suspension test shows predictive validity for negative symptoms of schizophrenia.

## References

[1] Thaker GK, Carpenter WT (2001). Advances in schizophrenia. *Nature Medicine*.

[2] Reus VI (2008). *Mental Disorders*.

[3] Keshavan MS, Diwadkar VA, Montrose DM, Rajarethinam R, Sweeney JA (2005). Premorbid indicators and risk for schizophrenia: a selective review and update. *Schizophrenia Research*.

[4] Nuechterlein KH, Dawson ME (1984). Information processing and attentional functioning in the developmental course of schizophrenic disorders. *Schizophrenia Bulletin*.

[5] Laurent A, Saoud M, Bougerol T (1999). Attentional deficits in patients with schizophrenia and in their non-psychotic first-degree relatives. *Psychiatry Research*.

[6] McLaren S, Cookson JC, Silverstone T (1992). Positive and negative symptoms, depression and social disability in chronic schizophrenia: a comparative trial of bromperidol and fluphenazine decanoates. *International Clinical Psychopharmacology*.

[7] Breier A, Buchanan RW, Kirkpatrick B (1994). Effects of clozapine on positive and negative symptoms in outpatients with schizophrenia. *American Journal of Psychiatry*.

[8] Steru L, Chermat R, Thierry B, Simon P (1985). The tail suspension test: a new method for screening antidepressants in mice. *Psychopharmacology*.

[9] Porsolt RD, Bertin A, Jalfre M (1977). Behavioral despair in mice: a primary screening test for antidepressants. *Archives Internationales de Pharmacodynamie et de Therapie*.

[10] Noda Y, Yamada K, Furukawa H, Nabeshima T (1995). Enhancement of immobility in a forced swimming test by subacute or repeated treatment with phencyclidine: a new model of schizophrenia. *British Journal of Pharmacology*.

[11] Noda Y, Mamiya T, Furukawa H, Nabeshima T (1997). Effects of antidepressants on phencyclidine-induced enhancement of immobility in a forced swimming test in mice. *European Journal of Pharmacology*.

[12] Bleich A, Brown SL, Kahn R, Van Praag HM (1988). The role of serotonin in schizophrenia. *Schizophrenia Bulletin*.

[13] Meltzer HY (1989). Clinical studies on the mechanism of action of clozapine: the dopamine-serotonin hypothesis of schizophrenia. *Psychopharmacology*.

[14] Kapur S, Remington G (1996). Serotonin-dopamine interaction and its relevance to schizophrenia. *American Journal of Psychiatry*.

[15] Gelenberg AJ, Doller JC (1979). Clozapine versus chlorpromazine for the treatment of schizophrenia: preliminary results from a double-blind study. *Journal of Clinical Psychiatry*.

[16] Castelao JF, Ferreira L, Gelders YG, Heylen SLE (1989). The efficacy of the D_2_ and 5-HT_2_ antagonist risperidone (R 64 766) in the treatment of chronic psychosis. An open dose-finding study. *Schizophrenia Research*.

[17] Lieberman JA, Kane JM, Johns CA (1989). Clozapine: guidelines for clinical management. *Journal of Clinical Psychiatry*.

[18] Angrist B, Rotrosen J, Gershon S (1980). Differential effects of amphetamine and neuroleptics on negative vs. positive symptoms in schizophrenia. *Psychopharmacology*.

